# Human Rights in Hospitals: an End to Routine Shackling

**DOI:** 10.1007/s11606-023-08584-8

**Published:** 2024-01-02

**Authors:** Neil Singh Bedi, Nisha Mathur, Judy D. Wang, Avital Rech, Nancy Gaden, George J. Annas, Sondra S. Crosby

**Affiliations:** 1 Boston University Chobanian and Avedisian School of Medicine, Boston, MA USA; 2 Center for Health Law, Ethics, and Human Rights, Boston University School of Public Health, Boston, MA USA; 3 Boston Medical Center, Boston, MA USA

**Keywords:** shackling, restraints, incarceration, human rights, hospital policy

## Abstract

Medical students (NSB, NM, JDW) spearheaded revision of the policy and clinical practice for shackling incarcerated patients at Boston Medical Center (BMC), the largest safety net hospital in New England. In American hospitals, routine shackling of incarcerated patients with metal restraints is widespread—except for perinatal patients—regardless of consciousness, mobility, illness severity, or age. The modified policy includes individualized assessments and allows incarcerated patients to be unshackled if they meet defined criteria. The students also formed the Stop Shackling Patients Coalition (SSP Coalition) of clinicians, public health practitioners, human rights advocates, and community members determined to humanize the inpatient treatment of incarcerated patients. Changes pioneered at BMC led the Mass General Brigham health system to follow suit. The Massachusetts Medical Society adopted a resolution authored by the SSP Coalition, which condemned universal shackling and advocated for use of the least restrictive alternative. This will be presented to the American Medical Association in June 2024. The Coalition led a similar effort to coauthor a policy statement on the issue, which was formally adopted by the American Public Health Association in November 2023. Most importantly, in an unprecedented human rights victory, a BMC patient who was incarcerated, sedated, and intubated was unshackled by correctional officers for the purpose of preserving human dignity.

## INTRODUCTION

Routine shackling of incarcerated patients with metal restraints is widespread in hospitals across America, with the exception of perinatal patients. Despite harmful effects on patients and national attention to health equity, incarcerated patients are routinely shackled regardless of consciousness, mobility, illness severity, or age.^1,2^ A large cohort study (*n* = 1078) of Israeli hospitals found that 84% of incarcerated patients who have severely impaired mobility for medical reasons are shackled, nonetheless.^3^ The discriminatory and dehumanizing practice of routinely applying shackles to incarcerated patients exacerbates existing care disparities by violating both medical ethics and human rights principles. The 2018 federal *First Step Act* prohibited “the shackling of pregnant prisoners in federal custody, except in certain cases.”^4^ As the name of this criminal justice bill suggests, the ban did not entirely eliminate shackling and the practice continues to impact nonpregnant incarcerated patients.^5^

There is a common misconception among healthcare professionals that they cannot influence the use of shackles to restrain their patients. Protocols for shackling incarcerated patients can and must be changed to respect human dignity and health, while simultaneously providing safety in the workplace.^6–8^

## HARMS TO INDIVIDUALS

Shackling impacts physical health in several ways. In the hospital setting, restraints can result in skin breakdown, circulation compromise, compressive neuropathies, fractures, increased fall risk, increased risk of delirium, and predisposition to severe vascular injury.^1,6,9,10^ Clinicians may be limited in their ability to perform a thorough physical examination.^1^

Clinician bias against the shackled patient may also harm the clinician-patient relationship. In fact, the presence of shackles negatively affects empathy, precipitates diagnostic skepticism, and elicits unsubstantiated fears of personal harm by the patient.^6^ Shackles have led to insensitive, inappropriate, neglectful, or abusive actions by staff or associated authority figures, which in turn evokes a response of fear in patients along with a loss of trust in the care team.^11^ These negative healthcare interactions further stress incarcerated patients’ post-carceral challenges within the healthcare system.^12^

## HUMAN RIGHTS VIOLATIONS

Routine shackling of incarcerated patients violates foundational international human rights principles, including those contained in the Universal Declaration of Human Rights, the International Convention on the Elimination of All Forms of Racial Discrimination, and the International Covenant on Civil and Political Rights.^13–15^ These principles are designed to protect human dignity and protect persons from discrimination and cruel, inhuman, and degrading treatment. Shackling patients who are critically ill or at the end of life is an affront to their human dignity and increases pain and suffering in this vulnerable time. Routine shackling violates the United Nations (UN) Standard Minimum Rules for the Treatment of Prisoners (The Nelson Mandela Rules)—the internationally accepted standard for the treatment of prisoners.^16^ Rule 48 addresses the use of restraints (see Box 1).

**Table:** 

**Box 1: UN Standard Minimum Rules (The Nelson Mandela Rules) Rule 48**
1. When the imposition of instruments of restraint is authorized in accordance with paragraph 2 of rule 47, the following principles shall apply: (a) Instruments of restraint are to be imposed only when no lesser form of control would be effective to address the risks posed by unrestricted movement; (b) (c) The method of restraint shall be the least intrusive method that is necessary and reasonably available to control the prisoner’s movement, based on the level and nature of the risks posed; Instruments of restraint shall be imposed only for the time period required, and they are to be removed as soon as possible after the risks posed by unrestricted movement are no longer present 2. Instruments of restraint shall never be used on women during labor, during childbirth and immediately after childbirth

In accordance with these Mandela Rules and the Charter of Fundamental Rights of the European Union, the head of the British prison service proclaimed that the “shackling of prisoners in hospital[s] should not occur,” emphasizing that “security is important, but it should never blind us to the overriding need for compassion and humanity.”^17,18^ In the Netherlands, chains are never used and handcuffs are used only in exceptional circumstances.^19^ Our work amended shackling practices to introduce a risk-stratified, individualized protocol for American hospitals that aligns with human rights principles better upheld by Western nations.^20,21^

## FROM CHANGING HOSPITAL POLICY TO LAUNCHING A NATIONAL MOVEMENT

We began by writing and circulating a petition to local hospital affiliates and community members to raise awareness and support.^22^ The petition then spread nationally, amassing 780 signatures across 129 institutions. The response demonstrated a consensus for change that enabled us to engage the hospital’s executive leadership in policy reform. We solicited input from key hospital stakeholders including nursing and medical staff, public safety, and general counsel, and collaborated with the local correctional facility to guide implementation. Identifying shared values for patient care helped generate feedback to balance concerns for safety and liability with human dignity. These partnerships resulted in the modification of hospital policy in February 2023. The core of the humanized policy is a protocol that allows for the removal of shackles from certain incarcerated patients. The policy outlines a schema for communication and decision-making among correctional facilities, hospital security, and the patient’s healthcare team.

The discourse around shackling practices reached beyond the walls of our hospital, inciting a national discussion about ending the practice of routine shackling of incarcerated patients. In parallel to modifying the policy at BMC, we also launched the Stop Shackling Patients Coalition (SSP Coalition)—a body of clinicians, public health practitioners, human rights advocates, and community members who share the goal of humanizing the inpatient treatment of incarcerated patients. SSP Coalition has grown into a diverse task force and learning collaborative that meets to empower more than a dozen healthcare institutions from across the USA to change their shackling policies.

## A NOVEL PROTOCOL

**Figure 1:**
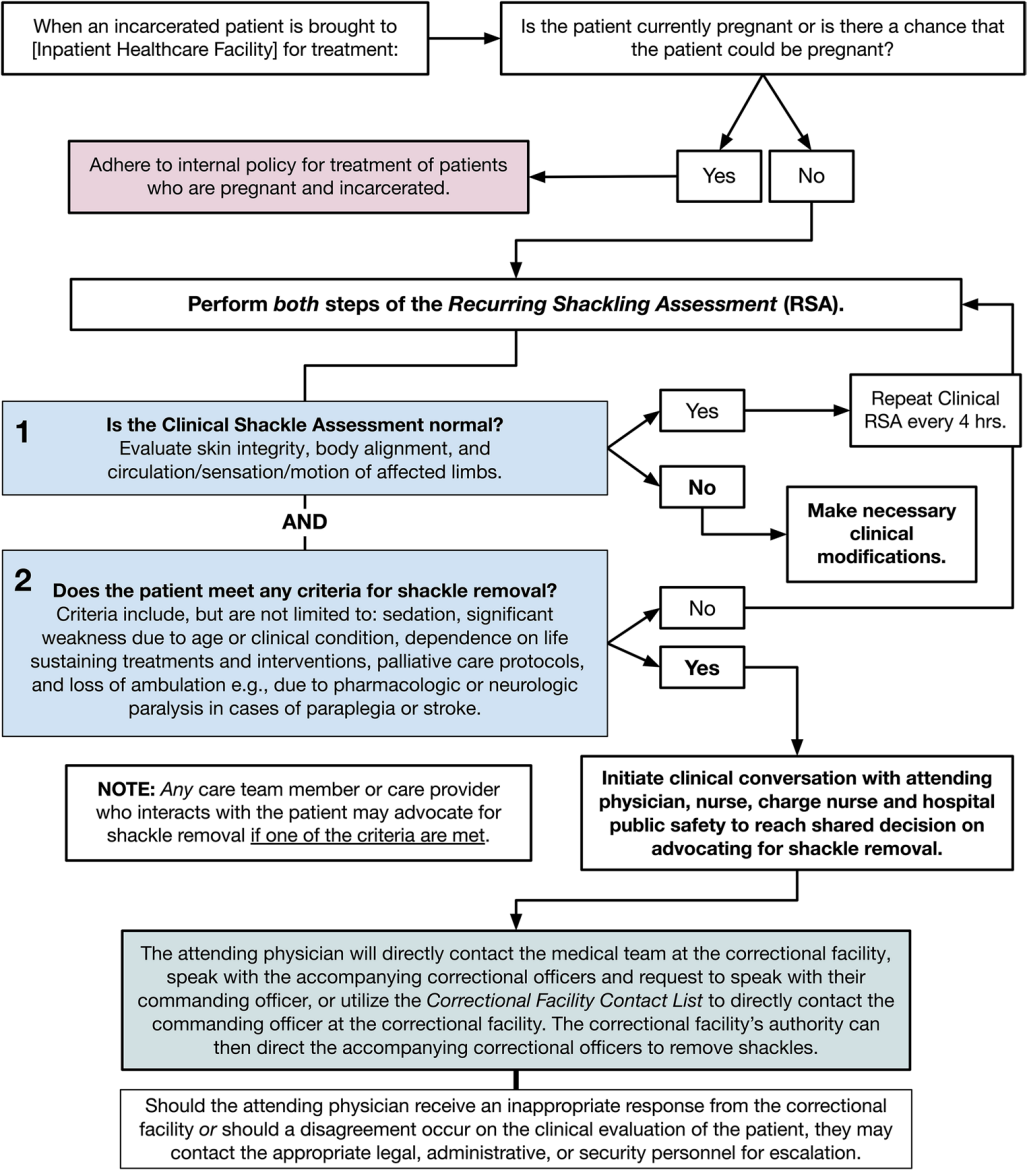
Generalizable protocol to supplement existing hospital policies for the care of incarcerated patients. This flowchart depicts the generalizable model that can be followed by any healthcare institution for the removal of shackles.

Though this protocol was designed and first implemented at Boston Medical Center, the goal is to disseminate this policy across US hospitals. For that reason, a *generalizable protocol* for shackle removal was designed that could be incorporated into existing hospital policies for the care of incarcerated patients (Fig. 1). The protocol parallels existing clinical assessments of any patient who is restrained in the hospital for medical or behavioral reasons and is incorporated into the electronic health record (EHR). The EHR identifies incarcerated patients and prompts a member of the healthcare team to perform a *Recurring Shackle Assessment*. This functions to determine if a shackled, incarcerated patient meets any *Special Circumstances* for shackle removal (Fig. 1). These include but are not limited to the following: sedation, significant weakness due to age or clinical condition, dependence on life-support, end-of-life care, or paralysis for any reason. If the patient meets any Special Circumstance, the EHR protocol prompts the healthcare team to determine whether shackle removal is appropriate.

If appropriate, care team members first notify hospital public safety. The attending physician directly calls the medical team at the correctional facility, requests the accompanying correctional officers to contact their commanding officer, or utilizes the Correctional Facility Contact List—an appendix to the hospital policy that includes direct points of contact at each local correctional facility—to communicate with the commanding officer at the facility. The supervisor can then direct the accompanying correctional officers to remove shackles. If there is a disagreement between the care team and the correctional facility about shackle removal, the care team follows an appeal process, escalating the request to hospital public safety leadership.

This protocol identifies incarcerated patients who may be safely unshackled and provides a framework for restraint removal while ensuring safety. It can be integrated into existing policy systems, EHR flowsheets, and healthcare workflows, while also providing individualized care for incarcerated patients (see Box 2).

**Table:** 

**Box 2: Excerpt from the updated policy on the *Care of Incarcerated Patients* –** **Boston Medical Center**
In addition to the Care of Prisoners documentation by nurses, any member of the incarcerated person’s interdisciplinary healthcare team shall assess the incarcerated person’s health status to determine if Special Circumstances are present for shackle removal or modification to the least restrictive alternative. This Recurring Shackle Assessment (RSA) shall be documented every twelve hours. If the patient meets criteria for Special Circumstances, the healthcare team will determine whether the patient is eligible for Compassionate Shackle Removal (See “Compassionate Shackle Removal for Hospitalized Patients). If Special Circumstances are discovered at any other time than during a RSA, restraint removal or modification can also occur * Compassionate Shackle Removal for Hospitalized Patients* Compassionate Shackle Removal is a concept where, in exceptional cases, the patient’s clinical team may request the patient’s custodial agency to recommend removal of handcuffs and shackles while the patient is receiving care at BMC. Consideration for these requests should be discussed with the patient Care Team to evaluate the patient’s physical ability to cause harm to themselves or others, or attempt escape If the Care Team agrees the patient meets the criteria for compassionate shackle removal, the Attending Physician is encouraged to advocate to the custodial agency for the removal of the shackling devices outlined in Fig. 1

Both policy and practice have begun to change. Changes pioneered at BMC led the Mass General Brigham health system to follow suit. The Massachusetts Medical Society adopted a resolution authored by the SSP Coalition, which condemned universal shackling and advocated for the use of the least restrictive alternative.^23^ This will be presented at the American Medical Association's annual meeting in June 2024. The Coalition led a similar effort to coauthor a policy statement on the issue, which was formally adopted by the American Public Health Association in November 2023. Most importantly, in an unprecedented human rights victory, a BMC patient who was incarcerated, sedated, and intubated was unshackled by correctional officers for the purpose of preserving human dignity. The next steps include continuing staff education about the modified policy and EHR documentation. Ongoing and iterative improvement and evaluation will be important for sustainable implementation.

We encountered obstacles in parsing patient and physician rights, entrenched clinical practices, stigma, and culpability. Determining who can request the modification or removal of shackles, who wields the practical authority to approve or deny such requests, and identifying and engaging all stakeholders was challenging. This stems from limited interactions, often through third-party healthcare or security contractors, between the correctional system and hospital-based medical care. Clearing the haze required us to identify written policies wherever possible, collaborate with colleagues from across clinical specialties and with hospital administration, and work directly with correctional facility leadership. We overcame barriers to change in a stepwise process that can serve as a model for other initiatives at the intersection of human rights and medicine.

The harmful and discriminatory routine shackling of incarcerated patients continues to occur in the American healthcare system. Though there is complexity in balancing patient dignity, hospital safety, and correctional responsibility, healthcare professionals cannot be bystanders to human rights violations in their own hospitals*.* It is our hope that healthcare institutions across the nation will be inspired to examine and challenge their shackling practices through concrete policy change. As healthcare professionals, we are obligated to expose practices that perpetuate harm and work to humanize the care of *all* patients.
